# Mechanical Performance of Corn Starch/Poly(Vinyl Alcohol) Composite Hydrogels Reinforced by Inorganic Nanoparticles and Cellulose Nanofibers

**DOI:** 10.3390/gels8080514

**Published:** 2022-08-18

**Authors:** Hiroyuki Takeno, Rina Shikano, Rin Kikuchi

**Affiliations:** 1 Division of Molecular Science, Graduate School of Science and Technology, Gunma University, Kiryu 376-8515, Gunma, Japan; 2 Gunma University Center for Food Science and Wellness, 4-2 Aramaki, Maebashi 371-8510, Gunma, Japan

**Keywords:** clay platelets, silica nanospheres, cellulose nanofiber, composite hydrogel, self-healing, corn starch, multi-crosslinking

## Abstract

We investigated the mechanical properties of corn starch (CS)/poly(vinyl alcohol) (PVA)/borax hydrogels reinforced by clay platelets, silica (SiO_2_) nanospheres, or cellulose nanofibers (CNFs). The effects of these reinforcing agents on the tensile properties of the hydrogels were quite different; the fracture stress of SiO_2_/CS/PVA/borax composite hydrogels increased with SiO_2_ concentration, whereas that of clay/CS/PVA/borax composite hydrogels was high at a low clay concentration but low at high clay concentrations; for CNF/CS/PVA/borax composite hydrogels, although the elastic modulus was highly enhanced by adding CNF, the fracture stress was very low because of the stress relaxation during the elongation. This result came from differences in the dispersibility of each filler and the reinforcing ability. These composite hydrogels were constructed by multi-crosslinking, such as hydrogen bonding between CS and PVA, CS and PVA crystals, complexation between borate and PVA (partly CS), and the crosslinking between each filler and polymer. The self-healing ability of SiO_2_ and clay composite hydrogels was examined. As a result, the SiO_2_/CS/PVA/borax composite hydrogels possessed an excellent self-healing ability, whereas the clay/CS/PVA/borax composite hydrogels had a poor self-healing ability.

## 1. Introduction

Recently, interest has been increasing in the use of bio-based polymer materials to reduce the consumption of fossil fuels. Starch is a bio-based polymer composed of a mixture of amylose and amylopectin, and it is mainly used as a thickening, gelling agent in the industrial field. Starch can be crosslinked with chemical crosslinkers, such as glutaraldehyde and epichlorohydrin [1,2], or physically by retrogradation (the recrystallization of starch) [3]. However, the mechanical properties of its hydrogels are usually poor; it is particularly difficult to produce highly stretchable hydrogels. A blending of starch and flexible polymers, such as poly(vinyl alcohol) (PVA), is effective for the improvement of mechanical performance. Recently, Qin et. al. reported that corn starch (CS)/PVA hydrogels using borax as a crosslinker were highly stretchable, and they used the freezing–thawing method to improve the mechanical performance [4]. Although the hydrogels exhibited very high elongation (>2000%), the tensile strength (12.5 kPa) was quite small.

Inorganic nanoparticles or nanofibers have, to date, been used for the mechanical reinforcement of polymer hydrogels. The incorporation of clay platelets, silica nanospheres, and nanofibers (such as cellulose nanofibers and chitosan nanofibers) into polymer hydrogels has been found to be effective in enhancing mechanical strength [5,6,7,8]. Clay platelets act as a multifunctional crosslinker that is capable of absorbing a lot of polymer chains on one clay platelet so that tough composite hydrogels can be produced [9,10,11,12]. Recently, we found that PVA/borax composite hydrogels using clay platelets or silica nanospheres as a filler are highly stretchable and robust [13]. Additionally, we reported that CNF/PVA/borax gels also had high extensibility and mechanical robustness [14,15]. However, a large amount of PVA (20 wt% or 30 wt%) was necessary for the preparation of these composite hydrogels. In this study, to lessen the amount of PVA derived from petrochemicals, we produced CS/PVA/borax composite hydrogels reinforced by clay platelets, SiO_2_ nanospheres, or CNFs. The influence of each filler on the mechanical properties is discussed from a structural point of view.

## 2. Results and Discussion

### 2.1. Mechanical Properties of SiO_2_/CS/PVA/Borax, Clay/CS/PVA/Borax, and CNF/CS/PVA/Borax Composite Hydrogels

**Figure 1:**
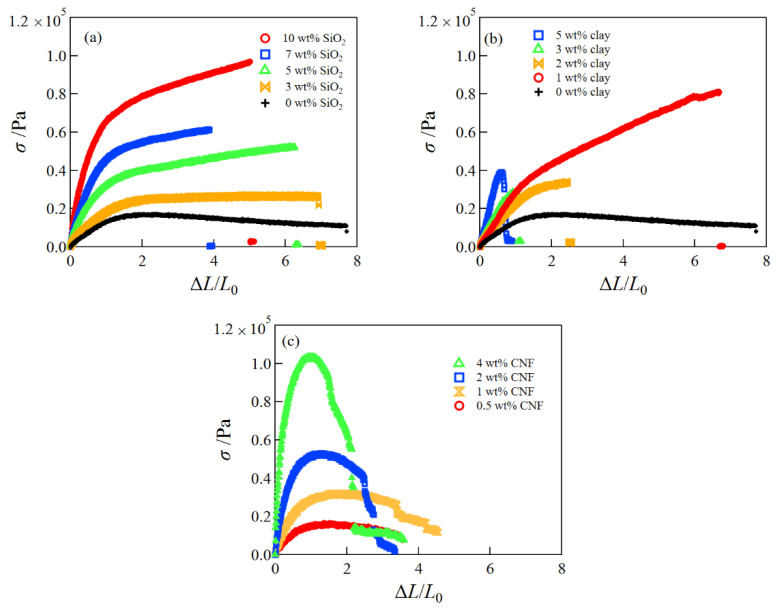
Typical tensile stress–strain curves of SiO_2_/CS/PVA/borax hydrogels at different SiO_2_ concentrations (**a**), clay/CS/PVA/borax hydrogels at different clay concentrations (**b**), and CNF/CS/PVA/borax hydrogels at different CNF concentrations (**c**).

Figure 1 presents the typical stress–strain curves of (a) SiO_2_/CS/PVA/borax composite hydrogels, (b) clay/CS/PVA/borax composite hydrogels, and (c) CNF/ CS/PVA/borax composite hydrogels. The tensile stress of the SiO_2_/CS/PVA/borax composite hydrogels increased with the increase in SiO_2_ content, whereas the mechanical performance of the clay/CS/PVA/borax composite hydrogels became poor at high clay concentrations. Although the tensile strength of the CNF/CS/PVA/borax composite hydrogels greatly increased with the increase in CNF content, the stress drastically decreased at larger strains; i.e., stress relaxation occurred.

**Figure 2:**
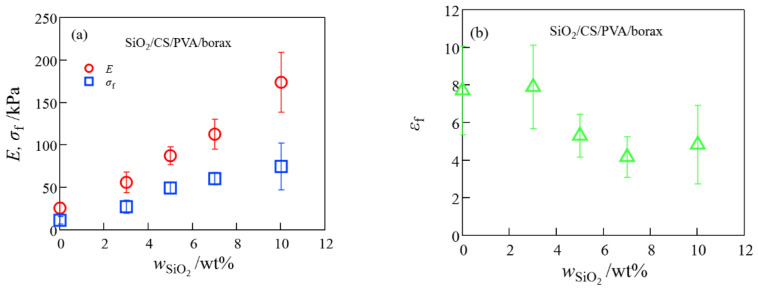
The Young’s modulus and the fracture stress (**a**) and the fracture strain (**b**) of SiO_2_/CS/PVA hydrogels at different SiO_2_ concentrations.

**Figure 3:**
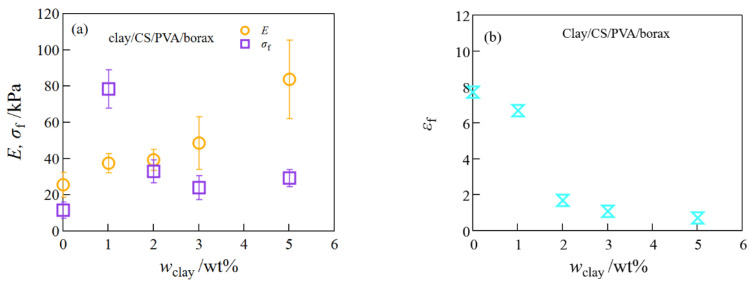
The Young’s modulus and the fracture stress (**a**) and the fracture strain (**b**) of the clay/CS/PVA hydrogels at different clay concentrations.

The mechanical performance of the SiO_2_/CS/PVA/borax composite hydrogels monotonously increased with SiO_2_ concentration under comparatively high elongation, whereas the mechanical performance of the clay/CS/PVA/borax composite hydrogels was high at a low clay content but low at a high clay content (Figure 2 and Figure 3). These different mechanical behaviors between SiO_2_/CS/PVA/borax and clay/CS/PVA/borax composite hydrogels are similar to those of SiO_2_/20 wt% PVA/borax and clay/20 wt% PVA/borax hydrogels reported in a previous paper [13], where the different mechanical properties between the SiO_2_/PVA/borax and clay/PVA/borax composite hydrogels came from differences in (i) the dispersibility of their nanoparticles and (ii) their functionalities; clay particles had more crosslinking points per particle than did SiO_2_ particles.

**Figure 4:**
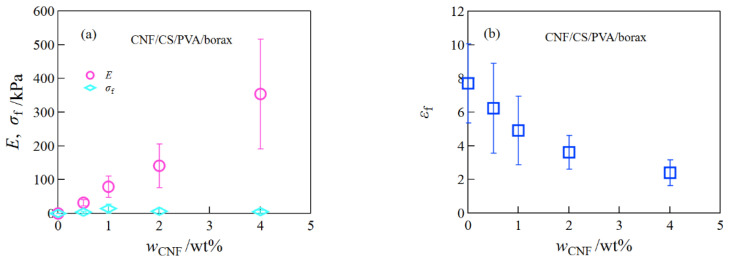
The Young’s modulus and the fracture stress (**a**) and the fracture strain (**b**) of CNF/CS/PVA hydrogels at different CNF concentrations.

For the CNF/CS/PVA/borax composite hydrogels, the increase in Young’s modulus with CNF concentration was much higher than that for the SiO_2_/CS/PVA/borax and clay/CS/PVA/borax composite hydrogels, but the fracture stress became quite low due to stress relaxation (Figure 4). This result suggests that the crosslink between CNF and the polymer network is quite weak.

### 2.2. FT-IR Measurements

**Figure 5:**
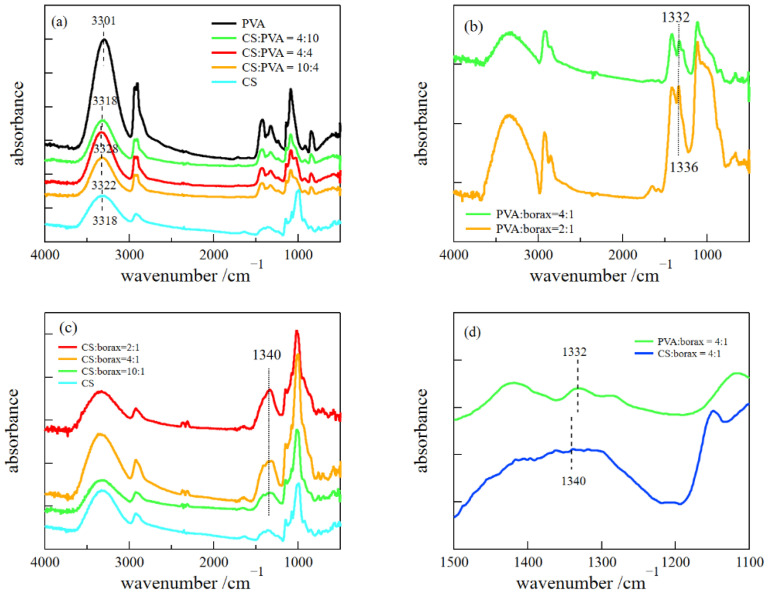
FT-IR spectra of CS/PVA (**a**), PVA/borax (**b**), and CS/borax (**c**), and a comparison between the spectra of PVA/borax and CS/borax (**d**).

We performed FT-IR measurements to examine the interactions between constituents (Figure 5). The characteristic bands around ~3300 cm^−1^ correspond to the stretching vibration mode of the hydrogen-bonded hydroxyl groups. The peak positions shifted to higher wavenumbers by blending PVA with CS, indicating that the hydrogen bonds between CS and PVA are weaker than those between pure polymers. The characteristic bands observed at ~1330 cm^−1^ correspond to the asymmetric stretching vibration of B-O-C, indicating the complexation between hydroxyl groups and borate [16,17,18].

Similarly, the characteristic band of CS/borax was observed at ~1340 cm^−1^ (Figure 5c). However, the peak was not clear compared to that of PVA/borax (Figure 5d), which suggests that the complexation between CS and borate tends to be less formed compared to that of PVA and borax.

### 2.3. Synchrotron SAXS/WAXS Measurements

**Figure 6:**
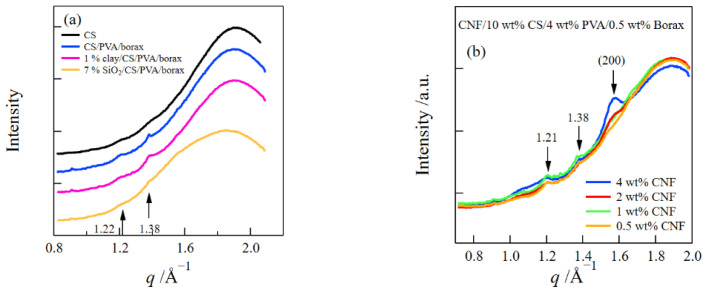
WAXS profiles of 10 wt% CS aqueous solution and CS/PVA/borax, 1 wt% clay/CS/PVA/borax, and 7 wt% SiO_2_/CS/PVA/borax composite hydrogels (**a**), and WAXS profiles of CNF/CS/PVA/borax composite hydrogels at different CNF concentrations (**b**).

Figure 6a depicts the WAXS profiles of the 10 wt% CS aqueous solution and the CS/PVA/borax, clay/CS/PVA/borax, and SiO_2_/CS/PVA/borax hydrogels. The peak at *q* = 1.38 Å^−1^ was assigned to the overlapping of (101) and ($10\bar{1})$ reflections of PVA crystals [14,19]. Additionally, a small shoulder was observed at *q* = 1.22 Å^−1^, which was assigned to the reflection from the A-type crystal of starch [20,21]. These results indicate that PVA and CS crystals exist in the composite hydrogels.

Figure 6b presents the WAXS profiles of the CNF/CS/PVA/borax composite hydrogels at various CNF concentrations. In addition to the scattering peaks of the CS and PVA crystals, another peak was observed at *q* = 1.57 Å^−1^, and the peak intensity increased with the increase in CNFs, which corresponds to the (200) reflection of cellulose I_β_ crystals [14,22,23].

**Figure 7:**
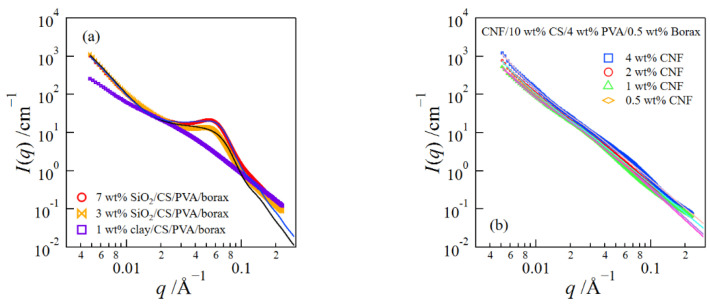

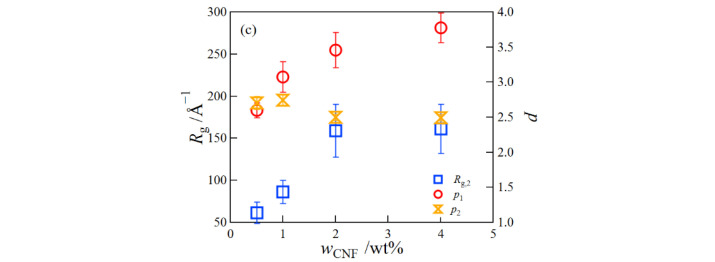
SAXS profiles of clay/CS/PVA/borax and SiO_2_/CS/PVA/borax composite hydrogels (**a**), SAXS profiles of CNF/CS/PVA/borax composite hydrogels at different CNF concentrations (**b**), and the parameters obtained by a fitting analysis (**c**).

Figure 7a shows the SAXS profiles of the SiO_2_/CS/PVA/borax and clay/CS/PVA/borax composite hydrogels. The SAXS profiles of the SiO_2_/CS/PVA/borax composite hydrogels have a broad peak at *q* ≈ 0.058 Å^−1^, which comes from the interference between SiO_2_ nanospheres. The upturn of the SAXS intensity was observed at small *q*’s due to the inhomogeneous distribution of the nanospheres. However, such an upturn of the SAXS intensity at low *q*’s was not seen for the 1 wt% clay/CS/PVA/borax composite hydrogel.

The scattering curves of spherical particles are described by

(1) $$I\left(q\right)\sim P_{\mathrm{sphere}}\left(q\right)S\left(q\right)$$

with

(2) $$P_{\mathrm{sphere}}\left(q\right)\sim {\left[\frac{3\left\{\sin\left(qR\right)\right\}-qR\cos\left(qR\right)}{{\left(qR\right)}^{3}}\right]}^{2}$$

where *P*_sphere_ (*q*) and *S*(*q*) are the form factor and the structure factor, respectively [24,25]. The former corresponds to the intra-particle scattering, whereas the latter corresponds to the inter-particle scattering. As a model of *S*(*q*), we used the Percus–Yevick (PY) equation [26], which is described with the interaction radius *R*_HS_ and the volume fraction of spheres *ϕ*. In addition, we considered the Gauss distribution, with the mean radius *R*_ave_ and the standard deviation *σ*_sphere_ for the size heterogeneities of the spheres. The details of the PY equations are described elsewhere [27]. The upturn of the scattering intensity at low *q*’s cannot be expressed by the PY model. Therefore, we used the Debye–Bueche (DB) model for the upturn [28], considering the inhomogeneous distribution of the nanospheres,

(3) $$I_{\mathrm{DB}}\left(q\right)\sim \frac{1}{{\left(1+\xi_{\mathrm{DB}}^{2}q^{2}\right)}^{2}}$$

where *ξ*_DB_ represents the correlation length of the inhomogeneous structure.

We analyzed the SAXS data of the SiO_2_/CS/PVA/borax composite hydrogels by combining the PY and DB models, i.e., using Equations (1)–(3). The obtained parameters are summarized in Table 1. The values of *R*_ave_ for both gels are close to that of the isolated SiO_2_ nanosphere (22.3 Å) evaluated in a previous paper [27]. This result suggests that the flocculation of the SiO_2_ nanospheres did not occur much in the composite gels. Additionally, *ξ*_DB_ did not change with the increase in the SiO_2_ concentration. Thus, the structural inhomogeneity in the gel did not increase at the high SiO_2_ concentration.

Next, we examined the structures of the CNF/CS/PVA/borax composite hydrogels at different CNF concentrations (Figure 7b). The scattering curves show the power-law behavior due to the scattering of CNFs [14] and have somewhat different behaviors at small and high *q*’s. Accordingly, we analyzed them using the Beaucage equations consisting of two structural levels (*R*_g,1_ > *R*_g,2_) [29,30,31].

(4) $$I\left(q\right)\;\sim \;G_{1}\exp\left(-\frac{q^{2}R_{g1}^{2}}{3}\right)+B_{1}{\left[erf\left(\frac{qR_{g,\;1}}{\sqrt{6}}\right)\right]}^{3p_{1}}q^{-p_{1}}\;\exp\left(-\frac{q^{2}R_{g,\;2}^{2}}{3}\right)+G_{2}\exp\left(-\frac{q^{2}R_{g,\;2}^{2}}{3}\right)+B_{2}{\left[erf\left(\frac{qR_{g,\;2}}{\sqrt{6}}\right)\right]}^{3p_{2}}q^{-p_{2}}$$

Here, *G*_*i*_ and *B*_*i*_ (*i* = 1 or 2) are the Guinier prefactor and the power-law prefactor, respectively. *R*_g,*i*_, and *p*_*i*_ represent the radius of the gyration of a structure at each structural level and the exponent of the power-law scattering, respectively. The parameters obtained by the fitting analysis are shown in Figure 7c. It was difficult to estimate the structural size *R*_g,1_ of the larger structure, because the scattering intensity at low *q* did not level off [32]. The values of *p*_1_ increased with the increase in CNF content, which suggests that the increase in the CNF content promoted the aggregation of CNFs. Thus, it was difficult to disperse CNFs at high concentrations.

### 2.4. Self-Healing

**Figure 8:**
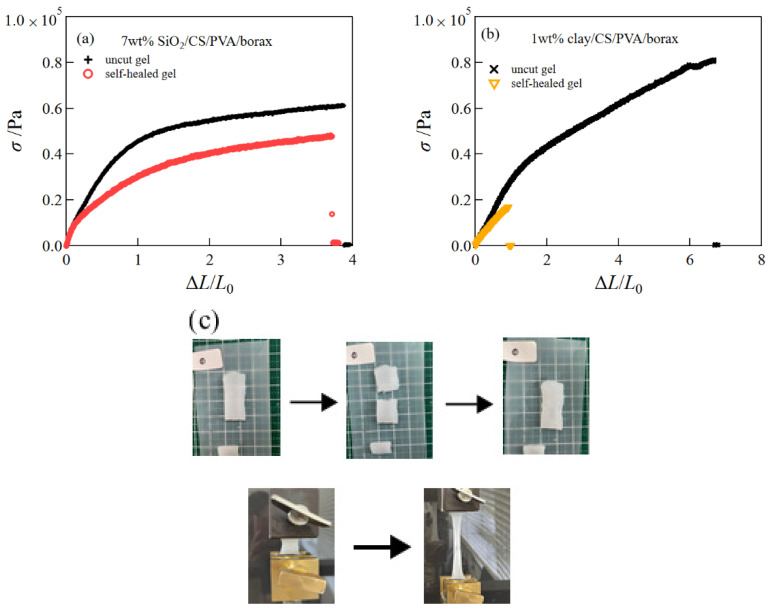
Representative tensile stress–strain curves of the self-healed gel and the uncut gel; 7 wt% SiO_2_/CS/PVA/borax hydrogels (**a**) and 1 wt% clay/CS/PVA/borax hydrogels (**b**). Pictures of a self-healed SiO_2_/CS/PVA/borax hydrogel (**c**).

In a previous study, we showed that CNF/PVA/borax hydrogels prepared using the freezing–thawing method possessed a good self-healing ability [14]. In this study, we investigated whether clay/CS/PVA/borax and SiO_2_/CS/PVA/borax hydrogels have a self-healing ability. To examine the self-healing ability of the hydrogels, tensile measurements for the self-healed hydrogel and uncut hydrogel were compared (Figure 8).

For the SiO_2_/CS/PVA/borax hydrogels, the tensile stress and the fracture strain of the self-healed sample were close to those of the uncut sample. For the clay/CS/PVA/borax hydrogels, although the tensile stress of the self-healed sample at small strains was close to that of the uncut sample, the extensibility was very low. The results of the tensile measurements for the self-healed hydrogels are summarized in Table 2. For comparison, the result of the self-healed hydrogels without inorganic nanoparticles is shown. The elastic modulus and fracture strain of the self-healed SiO_2_/CS/PVA/borax hydrogel attained 86% and 87% of those of the uncut hydrogel. Thus, SiO_2_/CS/PVA/borax hydrogel has a good self-healing capacity. However, the self-healing capacity of the clay/CS/PVA/borax hydrogel was inferior to that of the SiO_2_/CS/PVA/borax hydrogel. As shown in a previous study, the SiO_2_ nanoparticles formed a complexation with borax, whereas the clay particles did not [13]. The difference may be the cause of the different self-healing capacities of both composite hydrogels.

### 2.5. Comparison of Mechanical Properties of SiO_2_/CS/PVA/Borax, Clay/CS/PVA/Borax, and CNF/CS/PVA/Borax Composite Hydrogels

In this study, we examined the mechanical properties of CS/PVA/borax composite hydrogels using different fillers, namely, SiO_2_ nanospheres, clay platelets, and CNFs. The effect of spherical fillers on the elastic modulus of rubbers has been discussed using the Guth–Smallwood–Einstein equation [33,34,35].

(5) $$\frac{E_{c}}{E_{m}}=1+2.5\phi +14.1\phi^{2}$$

**Figure 9:**
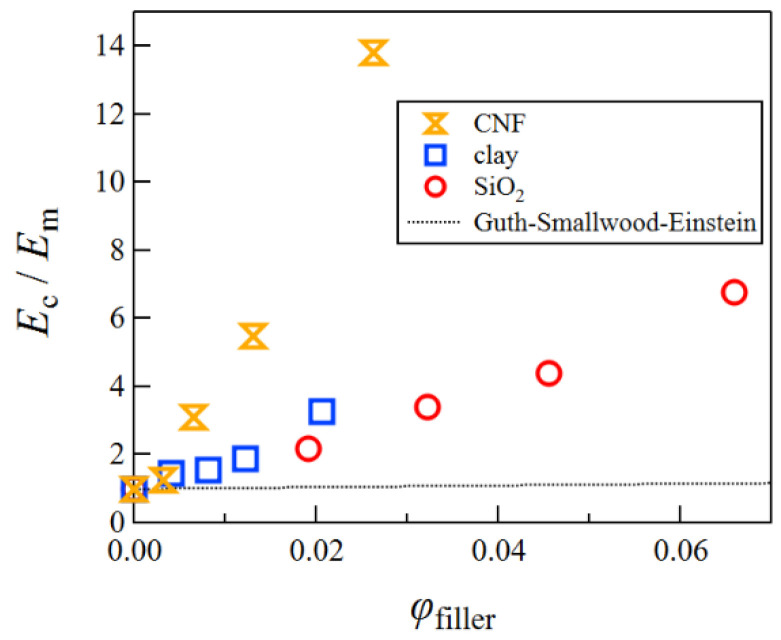
A plot of *E*_c_/*E*_m_ against the volume fraction of the filler for CS/PVA/borax composite hydrogels using various fillers.

Here, *ϕ* represents the volume fraction of the filler, whereas *E*_c_ and *E*_m_ are the elastic moduli of the rubber with and without the filler, respectively. We calculated *E*_c_/*E*_m_ for the composite hydrogels using SiO_2_ nanospheres, clay platelets, and CNFs as a filler (Figure 9). The values of *E*_c_/*E*_m_ for these composite hydrogels were much larger than the one expected from the Guth–Smallwood–Einstein equation, which expresses the hydrodynamic reinforcement of the filler. This difference may come from filler–filler interactions, filler–polymer interactions, or the anisotropy of the filler shape.

**Figure 10:**
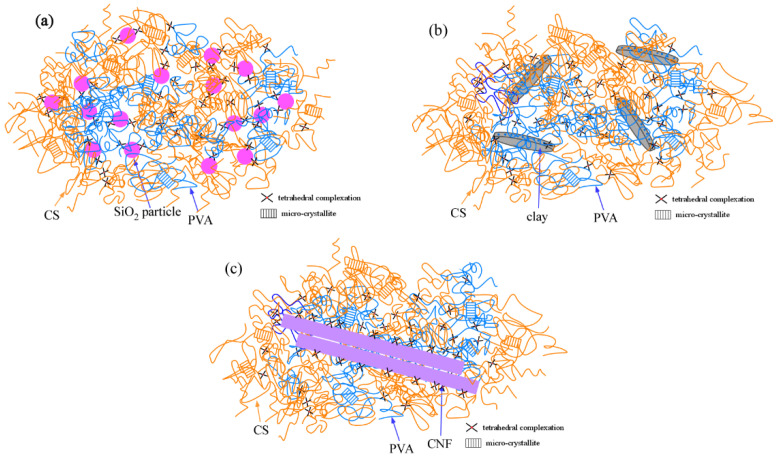
A schematic representation of SiO_2_/CS/PVA/borax (**a**), clay/CS/PVA/borax (**b**), and CNF/CS/PVA/borax (**c**) composite hydrogels.

A schematic representation of these composite hydrogels is presented in Figure 10. The reinforcement effect of CNF was the greatest, whereas that of the SiO_2_ nanospheres was the smallest. Thus, although the CNF/CS/PVA/borax hydrogels possessed a high reinforcing ability, the stress stored at larger strains was low due to the weak crosslink, such as the hydrogen bond between CNF and the network polymer [14]. Additionally, as revealed by the SAXS analysis, the increase in the CNF content caused the aggregation of CNFs, which lowered the mechanical performance. For the clay/CS/PVA/borax composite hydrogels, the mechanical performance was good at a low concentration; this was because the clay platelets were comparatively dispersed at a low clay concentration; additionally, the high multifunctionality enhanced the mechanical performance. To date, we have shown that the use of polymers with a molecular mass of more than a few million is necessary for the fabrication of tough clay/polymer composite hydrogels [36,37]. However, even if ultra-high molecular mass polymers are not used, the combined use of borax and clay platelets is effective for the production of tough composite polymer hydrogels. This is because the connection between the polymer and the crosslinker increases the length of the polymer network [13]. For the SiO_2_/CS/PVA/borax composite hydrogels, although the reinforcement effect of the SiO_2_ nanospheres is low, the dispersibility in aqueous systems is comparatively high. Therefore, it is possible to enhance the mechanical strength at high SiO_2_ concentrations without the large expense of stretchability. Thus, this study clarified that the type of filler significantly affects the mechanical performance of composite hydrogels.

## 3. Conclusions

We examined the mechanical and structural properties of the CS/PVA/borax using three kinds of fillers, namely, SiO_2_ nanospheres, clay platelets, and CNFs. Various experiments revealed that these composite hydrogels were constructed by the complexation between PVA (or CS) and borate, PVA and CS crystals, and a crosslink between the filler and the constituent polymer. The reinforcing effect of each filler on the mechanical properties of the CS/PVA/borax hydrogels depended on the dispersibility of the filler and the reinforcing ability. Although the addition of CNFs or clay to the CS/PVA/borax hydrogels caused the enhancement of the tensile strength at low concentrations of the filler due to the high reinforcing ability, the mechanical performance was lowered at high concentrations due to the increase in the inhomogeneity in the gel. However, the SiO_2_/CS/PVA/borax composite hydrogels showed a good mechanical performance even at high concentrations due to the high dispersibility in aqueous systems. Additionally, the SiO_2_/CS/PVA/borax composite hydrogels possessed an excellent self-healing ability.

## 4. Experimental Section

### 4.1. Materials

In this study, we used PVA, with a weight-average molecular weight of 94,000, which was estimated from a viscosity measurement, and hydrolysis higher than 99%, from Sigma-Aldrich, Tokyo, Japan, CS from Fujifilm Wako Pure Chemical Corporation, Tokyo, Japan, and sodium tetraborate decahydrate (borax) from Kanto Chemical Co. Inc., Tokyo, Japan. We used disk-shaped clay (Laponite XLG [Mg_5.34_Li_0.66_Si_8_O_20_(OH)_4_]Na_0.66_, RockWood Ltd., Tokyo, Japan) with a 26 nm diameter and 1 nm thickness [6], and we used amorphous silica nanospheres with a 4.5 nm diameter and a surface density of silanol groups of 4~6 /nm^2^ (Nissan Chemical Corporation, Tokyo, Japan). Tetrasodium pyrophosphate (TSPP) was used to prevent the aggregation of clay platelets. CNF (product name BinFi-s WMa) with a 5~10 µm length and 10~50 nm diameter was purchased from Sugino Machine Ltd., Toyama, Japan. The CNF had a degree of crystallinity of 0.49 [14].

### 4.2. Preparation of Composite Hydrogels

After a PVA aqueous solution was added to a CS aqueous solution, the mixture was heated in a water bath of ~90 °C and thoroughly stirred to dissolve the two solutions. For the preparation of clay/CS/PVA/borax or SiO_2_/CS/PVA/borax hydrogels, after a clay/TSPP aqueous dispersion or a SiO_2_ aqueous dispersion was mixed with the CS/PVA solution, borax was added to the clay/CS/PVA or SiO_2_/CS/PVA aqueous systems, and then the mixture was heated in a water bath at ~90 °C and thoroughly kneaded. For the preparation of CNF/CS/PVA/borax hydrogels, after CNF and borax were added to distilled water and then heated at ~90 °C to dissolve the borax, the mixture was mixed with an ultrasonic homogenizer (QSONICA model Q55). The CS/PVA solution was added to the CNF/borax suspension and then heated at ~90 °C. Afterward, the CNF/CS/PVA/borax mixture was thoroughly kneaded. The final compositions of CS, PVA, and borax were 10 wt%, 4 wt%, and 0.5 wt%, respectively.

### 4.3. Tensile Measurements

The prepared composite hydrogels were molded in a stainless spacer with 1 mm thickness, 10 mm length, and 15 mm width. Tensile measurements were performed at a stretching speed of 10 mm/min using a stretching machine (ORIENTEC TENSILE TESTER STM-20). The tensile stress was calculated using a cross-sectional area of an undeformed sample, and Young’s modulus *E* of the gel sample was obtained from a slope of the stress–strain curve at small strains. The average values of *E*, the fracture stress *σ*_f_, and the fracture strain *ε*_f_ were evaluated from at least four tensile tests. The fracture stress was determined from the value of stress at the point where the samples were fractured.

### 4.4. Fourier Transform Infrared Spectroscopy (FT-IR)

FT-IR measurements were carried out to characterize the hydrogels with JASCO FT/IR 4700. The samples for the FT-IR measurements were freeze-dried and were measured using the attenuated total reflection (ATR) method.

### 4.5. Synchrotron Simultaneous Small-Angle X-ray Scattering (SAXS)/Wide-Angle X-ray Scattering (WAXS) Measurement

Simultaneous synchrotron SAXS/WAXS experiments were performed on the beamline 6A of the Photon Factory at the High Energy Accelerator Research Organization in Tsukuba, Japan. X-rays with a wavelength of 1.5 Å were incident on the sample, and scattered X-rays were detected using two-dimensional detectors, PILATUS-1M for SAXS and PILATUS-100K for WAXS. The obtained two-dimensional images were circularly averaged to obtain the scattering intensity as a function of the magnitude of the wave vector *q* [38]. Here, *q* is defined by 4π sin(*θ*/2)/*λ* where *θ* and *λ* represent the scattering angle and wavelength of the X-ray, respectively. The scattering intensity obtained was thus corrected for the intensity of the incident X-ray beam, the background scattering intensity, and transmittance, and then it was reduced to absolute units [39].

### 4.6. Self-Healing

The self-healing ability of the clay/CS/PVA/borax and SiO_2_/CS/PVA/borax composite hydrogels was investigated. After the gel sample was cut in the middle of the sheet, the surfaces of the cut samples were placed in contact with each other. Afterward, the sample was placed at 25 °C for 20 h in an incubator. Tensile tests were conducted to examine the self-healing ability of the samples.

## Figures and Tables

**Table 1:** The result of the fitting analysis.

Sample	*R*_ave_/Å	*σ*_sphere_/Å	*R*_HS_/Å	*ϕ*	*ξ*_DB_/Å
3 wt% SiO_2_/CS/PVA/borax	32 ± 0.1	0.35 ± 0.002	40 ± 1.5	0.16 ± 0.007	349 ± 18
7 wt% SiO_2_/CS/PVA/borax	32 ± 0.007	0.35 ± 0.0004	45 ± 0.8	0.22 ± 0.005	340 ± 17

**Table 2:** Results of tensile measurements of self-healed hydrogels.

Samples	*E*/kPa	Recovery (*E*)/%	*ε*_f_	Recovery (*ε*_f_) /%
CS/PVA/borax (uncut)	25.7 ± 6.8	-	7.7 ± 2.4	-
CS/PVA/borax (self-healed gel)	19.4 ± 3.2	75	7.0 ± 2.0	91
clay/CS/PVA/borax (uncut)	37.5 ± 5.2	-	6.7 ± 0.3	-
clay/CS/PVA/borax (self-healed gel)	30.3 ± 2.3	81	1.1 ± 0.4	17
SiO_2_/CS/PVA/borax (uncut)	113 ± 17.9	-	4.2 ± 1.1	-
SiO_2_/CS/PVA/borax (self-healed gel)	96.9 ± 14.0	86	3.7 ± 1.2	87

## Data Availability

Data are contained within the article.
